# Supplementary material for: Dietary Fatty Acids Contribute to Maintaining the Balance between Pro-Inflammatory and Anti-Inflammatory Responses during Pregnancy
Source: Nutrients. 2023 May 23;15(11):2432. doi: 10.3390/nu15112432 (PMC10255171; doi:10.3390/nu15112432)
Supplement: Supplementary file 1 [file nutrients-15-02432-s001.zip › nutrients-2395551-supplementary.pdf]

Supplementary Figure S1

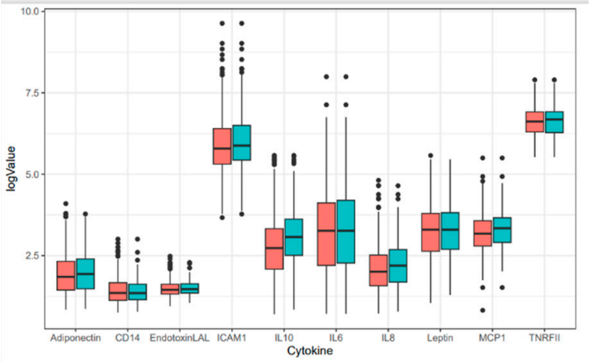

Supplementary Figure S1. Cytokine levels in the 250 women (orange box plots) and the all women tested for each cytokine (blue box plots).

Supplementary Figure S2

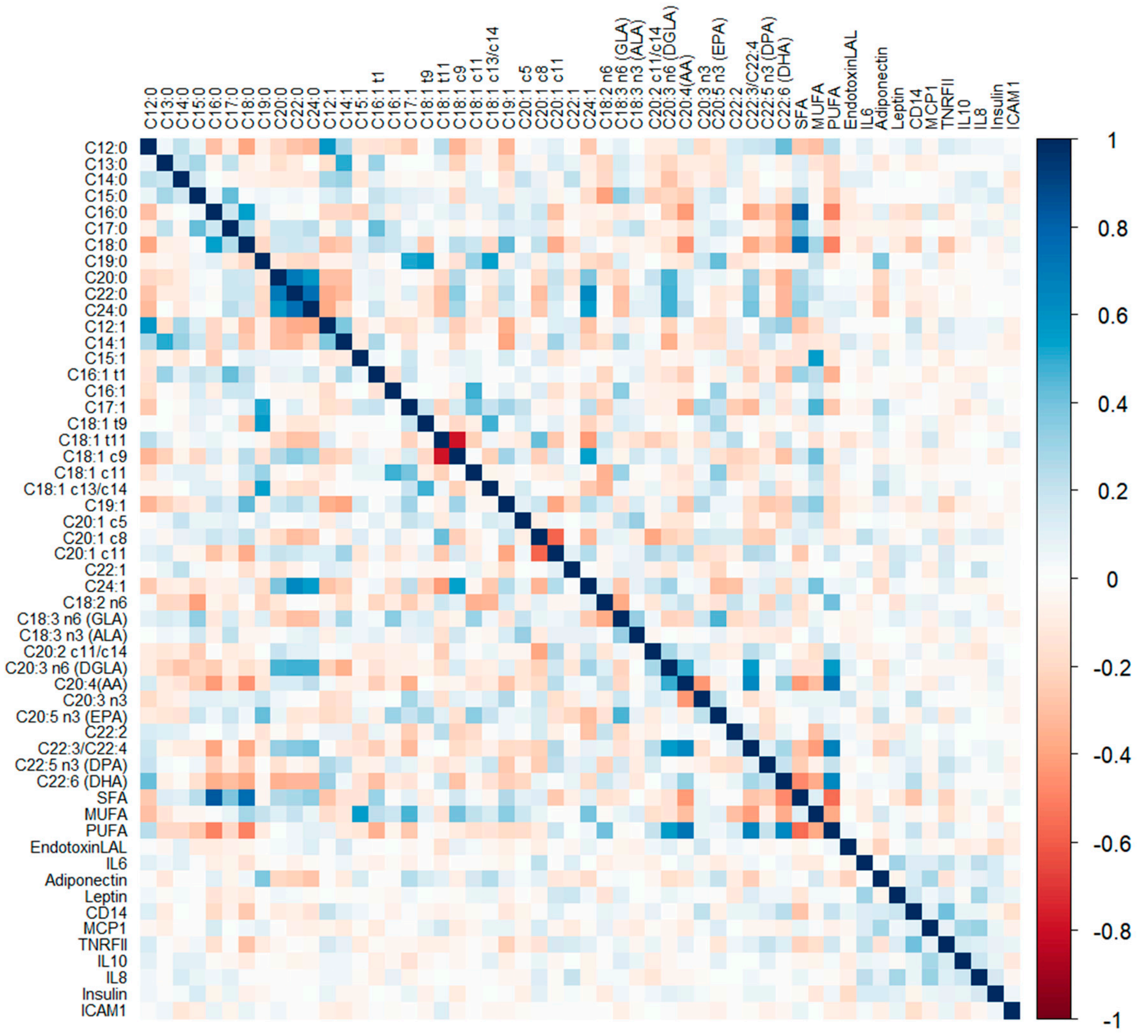

Supplementary Figure S2: Correlation plot of all the red blood cell membrane fatty acids (%) and circulating cytokines at 38 weeks of pregnancy.

Supplementary Table S1: mean values and standard deviation of fatty acids (%) at 38 weeks of gestation. AA, arachidonic acid; ALA, alpha-linolenic acid; DGLA, dihomo-gamma-linolenic acid; DHA, docosahexaenoic acid; DPA, docosapentaenoic acid; EPA, eicosapentaenoic acid; LA, linoleic acid; FAs, fatty acid methyl ester; MUFAs, monounsaturated fatty acids; PUFAs, polyunsaturated fatty acids; SFAs, saturated fatty acids. Total SFAs include: 12:0. 13:0. 14:0. 15:0. 16:0. 17:0. 18:0. 19:0. 20:0. 22:0. 24:0.

Fatty Acids (%)	
C12:0	1.11
C13:0	0.46
C14:0	1.07
C15:0	0.56
C16:0	20.02
C17:0	0.51
C18:0	13.81
C19:0	0.06
C20:0	0.37
C22:0	1.26
C24:0	3.81
C12:1	0.87
C14:1	0.80
C15:1	0.45
C16:1 t1	0.24
C16:1	0.68
C17:1	0.43
C18:1 t9	0.24
C18:1 t11	2.76
C18:1 c9	8.70
C18:1 c11	1.12
C18:1 c13/c14	0.31
C19:1	0.15
C20:1 c5	0.06
C20:1 c8	0.16
C20:1 c11	0.16
C22:1	0.09

C24:1	4.69
C18:2 n6	5.16
C18:3 n6 (GLA)	0.27
C18:3 n3 (ALA)	0.08
C20:2 c11/c14	0.21
C20:3 n6 (DGLA)	1.04
C20:4 (AA)	6.22
C20:3 n3	0.63
C20:5 n3 (EPA)	0.23
C22:2	0.52
C22:3/C22:4	1.35
C22:5 n3 (DPA)	0.84
C22:6 (DHA)	3.28
Total FAs	84.75
Total SFAs	43.06
Total MUFAs	21.95
Total PUFAs	19.78
Total n-3	1.78
Total n-6	12.7
Total Trans	3.2
